# Supplementary material for: Are carbon emissions trading and green financial instruments synergistic? -Comprehensive quantitative research based on content analysis
Source: PLoS One. 2024 Mar 7;19(3):e0298601. doi: 10.1371/journal.pone.0298601 (PMC10919601; doi:10.1371/journal.pone.0298601)
Supplement: S1 Data — (ZIP) [file pone.0298601.s001.zip › Data/地级市Z_F工作报告.zip!/╜¡╬≈/╥╦┤║╩╨/└┤╫╘╣1⁄2╓┌║┼í╛┬φ┐╦╩2╛▌═°í┐_2014.docx]

各位代表：

　　现在，我代表市人民政府向大会报告工作，请予审议，并请各位政协委员和其他列席人员提出意见。

　　一、2013年工作回顾

　　过去的一年，在省委、省政府和市委的正确领导下，我们紧紧依靠全市人民，解放思想，改革创新，较好地完成了市三届人大三次会议确定的目标任务，在建设文明幸福宜春的进程中迈出了坚实步伐。回首去年，我们难以忘怀，明月山机场建成通航，告别赣西地区无民用机场的历史；市经开区成功晋升国家级经济技术开发区，全市拥有更加坚实的发展平台。主要体现在以下方面：

　　综合实力有所提升。全年完成生产总值1387.1亿元，增长10.1%。财政总收入233.4亿元，增长16.5%，其中公共财政预算收入159.4亿元，增长20.4%。500万元以上固定资产投资1124.6亿元，增长21.3%。规模以上工业增加值631.9亿元，增长13.1%；新增规模以上工业企业110户，净增87户。实际利用外资5.3亿美元，增长11.6%。外贸出口总额18.3亿美元，增长22.8%。社会消费品零售总额404.8亿元，增长14.2%，增幅列全省第一。城镇居民人均可支配收入20871元，农民人均纯收入9115元，分别增长10.5%和13.2%。工业用电量96.1亿千瓦时，总量连续22个月居全省第一。新增金融机构贷款183亿元，列全省第三。中石化宜春分公司销售总额增幅列全省第一。县域经济竞相发展，财政总收入超20亿元县市4个、超15亿元7个，其中丰城财政总收入突破50亿元，居全国百强县第89位，较上年前移4位，樟树进入全国城市创新能力百强县市。

　　发展后劲明显增强。坚定不移主抓项目，完善重大项目协调推进机制，坚持每月召开现场办公会，研究解决突出问题140余个，促进项目快速落地。全市重大项目在库总数272个，其中10亿元以上项目116个。中心城区新火车站综合交通枢纽、多胜楼、科技之窗、宜阳森林公园等项目基本完工；杭长高铁、万宜高速、昌樟高速改扩建、丰厚一级公路、靖安洪屏抽水蓄能电站、市人民医院（北院）、市儿童医院等项目加快推进；碧桂园新城开发和昌栗、昌宁高速等项目顺利开工。奉新抽水蓄能电站选点正式获批。西凯新能源、合元科技、香港凯博等项目签约落户；中国瑞林、昆庆纺织等项目正式开工；宏宇能源、万国矿业、旺旺食品、科伦医疗器械等项目增资扩产；四特“两城”、紫宸科技、睿峰环保、奥其斯等项目投产见效；一批节能减排项目和环保处理设施推进建设或启用。

　　产业升级稳步推进。深入实施“四大战役”，产业升级提速增效。工业上水平。全市新增主营业务收入过亿元工业企业50户，纳税过千万元企业发展到228户。非公经济税收贡献率达到86.6%，列全省第一。以锂电新能源、绿色食品、生物医药为代表的战略性新兴产业快速发展，锂电产业产能挺进全国设区市十强，成功举办江西（宜春）锂电新能源产业合作推进会，生物医药产业主营业务收入占全省1/5，机械装备、绿色食品产业占全省比重超过1/10。全市工业园区实现主营业务收入2342亿元，列全省第三。樟树荣获中国药都称号，医药产业园获批国家新型工业化产业示范基地，被列为省级电子商务示范基地。丰城高新技术产业园及樟树、上高工业园获批重点省级园区。奉新被授予中国棉纺织名城，工业园区被评为中国中小企业创新服务先进园区。上高荣获全国食品工业强县。农业创佳绩。粮食、畜禽、油茶、竹、苗木花卉、有机（富硒）农产品等农业优势特色产业稳步发展，综合产值突破600亿元，粮食、油料、生猪等大宗农产品产量和粮食收购量均居全省第一，全国粮食生产先进县市个数占全省1/4，我市荣获全国高产油茶产业发展示范市，丰城、奉新、靖安分别荣获全国首批农业综合标准化示范县、中国绿色农业示范县、全国休闲农业与乡村旅游示范县。全市市级以上农业产业化龙头企业发展到280家，农民合作社总量达到3067家，全省农民合作社现场会在宜春召开，高安益农果业荣获全国优秀农民专业合作社。322座病险水库除险加固主体工程基本完工，9个小农水重点县工程、4座大型灌区续建配套与节水改造工程顺利完成，解决农村饮水安全32.3万人。率先在全省完成农村土地整治示范项目39.7万亩，荣获全省耕地保护成效显著设区市，奉新荣获全国国土资源节约集约模范县。城建显成效。全市新开工项目301个，续建188个，累计完成投资430亿元。预计全市城镇化率42%，比上年提高1.7个百分点，被省委、省政府授予新型城镇化先进市。投资10亿元的环城南路一年半内全线贯通，中心城区框架扩大近一倍，达到100平方公里，成为连接明月山机场和高铁站、促进区域联动发展和城南片区经济腾飞的快速通道。省级生态宜居城市考核中，中心城区排名第一，方科污水处理厂获全国十佳示范单位。选取19个示范镇（重点镇）积极推进镇村联动建设，得到省委领导高度肯定，其中15个列入全省百强中心镇。旅游服务业再升温。全市服务业增加值407.5亿元，占GDP比重提高1.9个百分点。成功举办第七届月亮文化旅游节，蝉联中国特色休闲城市，明月山硒泉养生万人泡脚活动创基尼斯世界纪录，被评为2013年中国旅游风云榜营销创新十佳。温汤集镇完成“六位一体”改造，明月山缆车升级改造、山顶观光小火车及樟树古海养生度假区等项目相继竣工。全年接待游客2145.3万人次，实现旅游综合收入155亿元，分别增长27.3%、25%。万民电子、林安物流等项目顺利推进。全年引进交通银行等4家股份制银行，上饶银行抓紧筹建。大力推广保险业，为群众理赔挽回经济损失8.5亿元。奉新华春色纺成功发行全省首单中小企业私募债。全省首家中外合资中银富登村镇银行落户宜丰。

　　改革开放不断深化。国有企事业单位改革深入推进，“营改增”试点顺利实施，县级部门预算改革全面开展，丰城纳入省县域经济发展综合改革试点县，率先在全省启动县级户籍制度全面放开试点。医药卫生体制改革稳步推进，全面实施国家基本药物制度，高安县级医院综合改革试点扎实开展。樟树药交会成功申报江西省首个商务部支持展会。文化体制改革步伐加快，国有文艺院团转企改制后活力增强。深入开展“招大引强年”活动，引进5000万元以上省外项目186个、工业园区在建项目501个，项目个数均列全省第一。与青海省海西、海东等地在循环经济和现代农业方面建立了战略合作关系。宜春海关申报工作进展顺利，高安、万载口岸作业区建设步伐加快。宜丰康替龙竹业建成全省首个企业内集装箱国检监管区。“质量兴市”工作取得新成效，新增中国驰名商标12件，总数达26件，新增数和总数连续两年居全省第一；新增省名牌产品19个、高新技术企业28家。樟树、万载等4个县市荣获全国科技进步先进县市。生态环境持续改善，新增国家、省级生态乡镇19个，靖安顺利通过国家级生态县验收。我市生猪养殖污染防治管理、靖安垃圾无害化处理做法在全省推广。

　　社会事业全面进步。全年新增可用财力70%以上用于保障和改善民生。新增城镇就业5.8万人，转移农村劳动力9.2万人。城乡养老、医保制度实现全覆盖。新农合筹资标准提高到人均340元，参合率达98%。在全省设区市成立首家残疾人创业信用担保机构。国家卫生城市长效管理工作得到国家爱卫办的充分肯定。全市基本建成保障性住房19741套、棚户区改造9014套、农村危房改造7680户。中心城区办理拆迁安置房权属登记8229宗，低保标准提高到每人每月410元，列全省第三。教育文化事业蓬勃发展，全市新建、改扩建中小学17所，新增学位1.8万个，城区中小学“大班额”问题进一步缓解。铜鼓荣获全省教育工作目标考核先进县。宜春学院获批教育部、卫生部第一批卓越医生教育培养计划试点。新增全国重点文物保护单位9处。采茶戏《阳台上的野菊花》获第五届江西艺术节玉茗花奖，《天宝往事》、《爱民太守况青天》获省优秀长篇小说奖，广播剧《守护春天》完成创作编排。第三届国际华文作家写作营在宜圆满闭营。禅宗文化“1+26”系列片拍摄进展顺利。全市荣登“中国好人榜”人数达到133人，上榜人数超过全省1/3，宜春再次承办全国关心关爱道德模范座谈会。皮祖强、郑向生分别获评第四届全国道德模范、全国道德模范提名奖，皮祖强、谭良才两人先进事迹在中央电视台《焦点访谈》栏目播出。曾凯被评为全国优秀公务员，并受到李克强总理亲切接见。认真贯彻现行生育政策。成功举办市第四届运动会，宜春籍运动员在第十二届全国运动会上获奖牌数居全省设区市第一，市体育局荣获全国群众体育先进单位，丰城被授予中国长寿之乡。完成县道升级改造、农村公路客运网络化连通工程、自然村通水泥路1300公里。实施自然村广播电视“村村通”工程1637个，率先在全省实现县县有数字影院。投资2.2亿元的铜鼓花山隧道基本贯通，彻底解决五个乡镇场近5万名群众出行难问题。中心城区一批开放式运动游园和3处幸福书屋投入使用，完成小街小巷改造28条，新建便民蔬果粮油直供超市20个、社区蔬菜直销店20个，改造升级农贸市场3个。加强重点行业和重要领域安全监管，安全生产形势稳定好转。畅通群众诉求渠道，重点信访问题和社会矛盾有效化解。市委、市政府政务微博“宜春发布”在新浪平台正式开通，荣获全省最佳形象奖。奉新“聘法律顾问、为群众解忧”的做法得到省委领导高度肯定并向全省推广。完成食品药品安全示范市创建。市物价局荣获全国价格工作先进集体。积极做好依法治理和应急管理工作，推进治安防控体系和“三网”建设，公众安全感测评稳居全省前三名，被评为全国社会管理综合治理优秀市。

　　与此同时，政府自身建设进一步加强。深入贯彻落实中央“八项规定”和省委若干规定，坚决反对“四风”，作风建设取得新成效。自觉接受市人大、市政协监督，认真执行市人大及其常委会的决定决议，虚心听取市政协的协商意见，办结市人大代表建议和政协提案288件，满意和基本满意率达100%。全面落实党风廉政建设责任制，深化行政审批制度改革和政务公开。主动公开各类政务信息12.1万条，组织开展在线访谈16期，邀请55名党政领导做客访谈。严格落实国务院“约法三章”，全面停止新建政府性楼堂馆所，财政供养人员、“三公”经费支出同比减少。加强审计、监察工作，公共权力运行、公共资源交易更加透明。民生资金监督管理做法在全省大会上作典型发言。国防后备力量建设、国家安全、外事侨务、民族宗教、涉台事务、邮政通信、烟草、盐业、气象水文、防震减灾、无线电管理、妇女儿童、统计、档案、扶贫、老龄和红十字会等工作均取得新成绩。

　　各位代表，在宏观形势趋紧、下行压力增大的情况下，我们沉着应对，主动作为，全市主要经济指标呈现稳步增长的态势，谱写了赶超发展的新篇章，这得益于省委、省政府和市委的正确领导，归功于全市上下的团结拼搏。在此，我代表市人民政府，向付出辛勤劳动的全市人民，向支持政府工作的人大代表和政协委员，向各民主党派、工商联、无党派、人民团体、驻宜部队、民兵预备役、武警官兵、公安民警和社会各界人士，向所有关心支持宜春发展的同志们、朋友们，致以崇高的敬意，表示衷心的感谢！

　　看到成绩的同时，我们也清醒地认识到，前进道路上面临不少困难和问题：发展基础不坚实，经济总量不大，人均值较低，赶超任务艰巨；城镇化水平偏低，中心城区辐射带动力较弱；强项目不够强，好项目不够好，大项目不够大，“航母”型特大项目尚需破题；改革攻坚力度、深度不够，安全维稳压力较大；一些政府工作人员服务意识和责任意识不强，发展环境有待进一步优化。我们将采取有力措施，切实加以解决。

　　二、2014年目标要求

　　2014年是全面贯彻落实党的十八届三中全会精神的关键之年，也是完整实施省委全会确定的“发展升级、小康提速、绿色崛起、实干兴赣”十六字方针的开局之年，做好全年政府工作，意义重大。新的一年，我们要把握形势，抢抓机遇：党的十八届三中全会对全面深化改革作出了总部署，改革主旋律进一步唱响，必将释放更多红利、拓展更大空间；省委、省政府大力实施赣西经济转型加快发展战略，制定了专门的支持政策，召开了高规格的现场推进会，对促进赣西地区转型发展，力度前所未有、机遇不可错过；随着明月山机场建成通航，杭长高铁即将通车，10县市区全部通高速，宜春全面迈入航空、高铁、高速公路时代，为我市承接产业转移提供了良好契机；市委审时度势，深入开展解放思想大讨论活动，全市上下谋发展、比实干、争先进的氛围更加浓厚。同时，我们也要冷静分析，应对挑战：省委全会后，全省新一轮发展势头强劲，竞争态势逼人，稍有松懈，标兵越跑越远，追兵越来越近；特别是由于企业用工紧张、工资上涨、融资成本提高，宜春与沿海发达地区的比较优势逐步弱化，招商竞争愈加激烈。我们要趋利避害，扬优成势，把问题看成任务，解决问题就是发展的机会；把困难看成使命，战胜困难就是前进的动力，牢牢掌握发展主动权。

　　今年政府工作的总体要求：全面贯彻党的十八大、十八届二中、三中全会以及省委十三届七次、八次全会精神，落实“稳中求进、改革创新”的基本要求，抢抓省委、省政府支持赣西转型发展机遇，全力推进产业升级，突出项目建设，做强中心城区，壮大县域经济，促进民生改善，确保主要经济指标继续保持赶超进位的好势头，确保社会和谐稳定，确保全面小康建设取得新成效。

　　全市经济社会发展主要预期目标：生产总值增长10.5%；财政总收入增长15%，其中公共财政预算收入增长17%；规模以上工业增加值增长13%；500万元以上固定资产投资增长21%；社会消费品零售总额增长13.5%；实际利用外资和外贸出口均增长10%；城镇居民人均可支配收入增长10.5%，农民人均纯收入增长11.5%；城镇化率提高1.7个百分点；城镇登记失业率控制在4.5%以内，人口自然增长率控制在8‰以内，居民消费价格指数控制在3.5%以内。

　　三、2014年工作重点

　　围绕实现“四大三确保”的目标，强化问题导向意识，着力培育项目气场，提升经略能力，树立底线思维，持续扩大有效投入，再掀全市赶超发展新高潮。突出抓好五个方面：

　　（一）紧扣改革创新这一主线

　　改革是加快发展的不竭动力。全面深化改革，必须以更大的决心和智慧，勇趟深水区、善打攻坚战，铲除拦路虎、扫清绊脚石。

　　深化重点改革。加快政府职能转变，三季度完成市县政府机构改革。分类推进事业单位改革，全面实行聘用和岗位管理制度。深化行政审批制度改革，探索负面清单管理模式，推进政府购买服务，将工作重心从事前审批到事后监管转变。探索组建“中介超市”，规范收费行为，切实降低企业成本。实施房建、水利、道路交通等行业公共资源网上交易。完善财税工作机制，推进财政预算公开试点，加大国库集中支付改革。深化农业农村改革，启动规范土地流转县乡试点，健全县、乡、村土地流转服务网络和交易平台，逐步建立农村产权交易市场，抓好樟树农村综合改革试验区建设和上高土地流转试点。深化各项社会事业改革，适时启动素质教育综合改革、义务教育均衡发展改革、中小学考试招生改革等试点。强化教师培养培训，推行公办学校校长、教师交流轮岗。深化医药卫生体制改革，推进社会资本办医改革，抓好高安、上高等县级公立医院综合改革试点。推进食品药品监督体制改革，完善食品药品监管体系。健全文化发展体制机制，深化国有经营性文化单位改革，大力培育文化骨干企业。完善市直行政单位国有资产统一经营和管理。继续做好国企改革扫尾工作，妥善处理宜工债务、长青厂破产资产处置及其他企业历史遗留问题。

　　壮大县域经济。以打造江西县域经济发展引领区为目标，优化生产力布局，完善考核评价机制，放手发展县域经济，努力构建中心城区和丰樟高“双核”驱动、其他县“块状隆起”的区域经济发展新格局。扩大县级发展权限，除国家明确规定应由市级审批、核准的项目外，原则上全部下放到县。支持丰樟高全面融入鄱阳湖生态经济区，主动对接南昌经济圈，引领县域经济发展。对其他县予以差异化支持，鼓励各县利用文化、生态等资源加快发展。袁州、上高、万载要各出奇招、各显其能，着力强产业、创特色，加强基础设施、产业配套对接，继续做大经济总量，形成宜春新“三驾马车”。靖安、奉新、宜丰、铜鼓要立足现有基础，发挥生态优势，大力发展旅游养生和新兴科技产业，打造宜春生态“四小虎”，形成赣西秀美风光带，建设成为湘鄂赣三省的旅游休闲后花园。

　　强化科技支撑。加快协同创新体系建设，推进产学研一体化，力争产学研合作项目达到200个，引进高科技项目20个，新增创新平台及创新团队15个。大力发展独具本土特色的高新技术产业集群，确保新增高新技术企业40家，产业增加值达到150亿元。加大品牌创建，力争新增国家地理标志保护产品1件、中国驰名商标4件，申请国家专利突破1400件。加快省锂电产品质量监督检测中心建设。积极推动企业上市，力争成功上市1家，进入辅导期1家，完成股改2家。落实人才优惠政策，实施招贤引智工程，力争培养引进各类高层次、高技能人才1000名以上，推进博士后流动站、院士工作站、国家地方联合工程研究中心等平台建设。

　　提升开放水平。深入开展“开放升级年”活动，更加注重招商选资，努力引进一批世界500强、跨国公司和知名民企。主动对接服务，支持央企兼并重组市内有条件的企业。积极引导宜商回乡创业。建成运营高安、万载口岸作业区，启动建设樟树水运口岸作业区、丰城集装箱海铁联运装卸站，力争开通危货通道。推进宜春铁路（锂电）口岸建设，积极打造航空物流中心、赣西出口加工区，对出口诚信企业实行检验检疫“绿色通道”和直通放行制度。争取宜春海关年内获批。进一步激活市场主体，大力发展非公有制经济，落实各项扶持政策，优化市场竞争环境，保护企业合法权益，力争新增私营企业2500户、个体工商户1.1万户。支持企业“走出去”，鼓励优势企业赴境外从事农业综合开发、矿产勘探和投资并购。

　　（二）把握产业升级这一根本

　　坚持以创新发展引领产业升级，在经济转型中实现规模做大、质量提升、效益更好。

　　促进工业升级。立足产业基础、资源禀赋、市场前景和区位优势，围绕打造一批千亿产业、千亿园区、百亿企业目标，科学制定产业规划，各县市区做大做强主导产业2 - 3个。加快发展新兴产业，重点推进锂电新能源、生物医药、绿色食品、先进装备制造等产业，力争主营业务收入占全市规模以上工业比重提高4 - 5个百分点。改造提升传统产业，利用高新技术和先进工艺，促进建材、纺织服装鞋革、化工电力能源等产业实现规模化、品牌化、新型化，争取申报并通过亿元以上技改项目30个。发挥龙头企业带动作用，推进企业上市和增资扩股，力争主营业务收入超50亿元企业达到4户，新增纳税超千万元以上企业40户。加快煤电、盐化工、废旧金属及塑料回收等产业发展，努力将丰城、樟树、高安打造成全省循环经济产业示范区，力争申报国家级循环经济试点园区或示范城市（县）1个以上。加快园区“三区一化”建设步伐，力争主营业务收入超500亿元园区1个、超400亿元园区4个，新获批重点省级工业园区1 - 2个，袁州、铜鼓获批筹建省级工业园区，全市工业园区主营业务收入突破3000亿元。

　　推动农业升级。创新农业生产经营体制机制，加快现代农业建设步伐，大力推进农业产业化，加速宜春从传统农业大市向农业强市迈进。突出抓好粮食、畜禽、油茶、竹、苗木花卉、有机（富硒）农产品等六大农业优势特色产业，力争全市农业产业化综合产值突破800亿元。积极培育农业产业化龙头企业，打造省级以上龙头企业100家。重点发展绿色农产品加工产业，支持上高创新中国成立家级绿色食品产业基地，培育一批在全国有竞争优势和影响力的绿色农产品品牌。加快现代农业示范园区建设，创新提升万载国家级现代农业示范区，支持袁州、丰城申报国家级现代农业示范区。创新农业经营方式，大力培育专业大户、家庭农场和农民合作社等新型经营主体，力争全市注册农民合作社达到3500家。坚持最严格的耕地保护和集约节约用地制度，提高土地利用效率，争取清理闲置或低效利用土地5000亩。实施“万村千乡”网络建设工程，恢复改造新建一批乡镇供销合作社。

　　加速旅游服务业升级。认真落实省委、省政府旅游强省意见，修编旅游发展总体规划，促进中心城区与县市区旅游资源共享，全面构建“双核三线四游”格局。充分挖掘温泉、禅宗文化、自然生态等特色旅游资源，加快旅游与文化深入融合。推进明月山景区年底前成功创新中国成立家5A级旅游景区，启动三爪仑景区创新中国成立家5A级旅游景区，推进奉新百丈山、宜丰东方禅文化园创新中国成立家4A级旅游景区，做好樟树筑卫城遗址申报世界遗产、禅宗文化申报世界遗产或国家级非物质文化遗产名录、温汤创建世界温泉名镇等前期工作，争取9月底完成禅宗文化“1+26”系列片制作并在中央电视台播出。珍惜和管控好明月山富硒温泉资源，切实做到科学开发、合理利用。办好第八届月亮文化旅游节，以“明月寄相思、畅叙中华情”为主题，着力把宜春打造成为两岸文化的交流胜地和海外华人的交流胜地。力争接待游客突破2700万人次，旅游综合收入超过200亿元。加快发展商贸流通、金融信息、电子商务等现代服务业，争创省级电子商务示范企业。抓好一批城市商贸综合体建设，推进市、县、乡农贸市场改造升级和便民蔬果粮油直供超市建设。以“空港”、“高铁”物流为增长点，建设一批综合物流园，培育壮大现有物流企业，努力实现服务业增加值超千亿元。进一步完善金融机构体系，做大金融总量，力争全年新增贷款170亿元，引进外埠银行1家，新设小额贷款公司2家、融资性担保机构1家。

　　（三）突出城乡发展这一关键

　　坚持全域统筹、整体谋划、加速提升，努力构建城乡一体、产城融合、生态宜居、社会和谐的城镇化发展格局。

　　做大做强中心城。当前，进一步做大做强中心城区、巩固提升宜春在赣西中心城市的地位已成共识、恰逢其时。随着航空、高铁时代的到来，中心城区交通条件大为改善，以前不中心，现在变成中心；在宜春不中心，在赣西却为中心；在江西不中心，在中部地区处于中部之心。连续七届月亮文化旅游节的成功举办，宜春的知名度、美誉度不断提升，人气、商气加速聚集。锂电、医药等新兴产业的崛起，一大批产业项目的落户，为叫响做强赣西中心城市增添了底气。积极对接省委、省政府打造新宜萍城镇密集带的重大部署，进一步优化航空、高铁、城南、明月大道两侧等重点区域规划布局，加快老城区改造，推进新城区建设。加大环城南路沿线开发力度，争取6月底启动320国道改线工程。突出抓好碧桂园新城开发、禅博园、北湖公园等在建工程，启动黄颇路片区棚户区改造、昌黎隧道、张家山跨高速公路立交桥等项目建设。进一步优化市区体制，强化市区一体化理念，支持袁州加快发展，合理确定中心城区“四区”管理权限，建立事权财权相匹配的管理体制；进一步明确政策导向，在财税、土地、项目、资金方面给予倾斜，引导各类生产要素向中心城区集中；进一步明确产业导向，加快现代旅游、总部经济、养老保健、商贸物流、电子商务、教育培训、锂电新能源、生物医药等新兴产业向中心城区集聚；进一步放宽入城门槛，鼓励外来人口和周边镇村农民在中心城投资入户。优先发展公共交通，确保群众出行便捷高效。大力实施“数字城管”工程，提高城市管理水平。按照创建健康城市的要求，巩固国家卫生城市成果，积极打造国家卫生城市升级版。规范房地产市场监管，促进房地产业健康有序发展。

　　统筹基础设施建设。完善综合交通网络，加快宜春新火车站综合交通枢纽、杭长高铁宜春段、煤运通道岳宜吉铁路、昌樟高速改扩建、万宜、昌栗、昌宁、昌傅至东乡高速公路、宜春至慈化公路等项目建设，推进昌吉赣客专、万铜高速、高安通用航空产业等项目建设，完善宜春明月山机场配套设施，增开连接国内主要城市航班。强化能源保障，加快靖安洪屏抽水蓄能电站等项目建设，推进中心城区四方井水库、奉新抽水蓄能电站、鹅婆岭水库、丰城龙头山水电枢纽工程、丰电三期、大唐国际上高电厂等项目前期工作。加大农田水利基本建设，继续推进病险水库除险加固、中小河流治理、小农水重点县和农村饮水工程建设。完善电子商务基础平台及服务系统，建立数字认证、网上支付、社会诚信体系和公共物流信息平台。

　　推进镇村联动。抓好省定新农村880个试点，集中力量实施19个示范镇（重点镇）镇村联动建设，力争6月底全面完成“新建十一个一、五个改造提质” 及集镇“六位一体”改造304个规定项目和96个联动村改造工作。加大跑项争资力度，积极采取土地“增减挂钩”运作模式，为镇村联动建设提供资金保障。加快各类规划编制，实现镇村规划全覆盖。合理规划产业结构和产业布局，着力发展特色小城镇产业。积极引导农民向集镇和中心村集居，提高集镇辐射力。巩固“三清一控”活动成果，严厉整治规划区内违规违法建房等突出问题。加强镇容镇貌管理，着力提升镇区品位、美化人居环境。

　　改善农村面貌。进一步做好新农村建设和农村扶贫帮扶到户工作，抓好国省道、高速公路等主要交通干道沿线村镇综合整治和改造提升。推进2226个行政村村庄规划编制，开展农村人居环境整治，实施清理垃圾、清洁水源和通自来水“两清一通”工程。继续开展广播电视“户户通”和电影下乡活动。加快推进县乡村公路建设，力争完成县道升级改造、农村公路客运网络化连通工程、自然村通水泥路800公里，自然村通畅率达到49%。

　　（四）坚守生态环保这一底线

　　把生态文明放在突出位置，实现绿色发展、循环发展、低碳发展，建设天蓝、地绿、水碧、气爽的美丽新宜春。

　　严抓生态保护。加速培育森林资源，全面禁止采伐天然阔叶林，切实提高森林覆盖率和活立木蓄积量。加强自然保护区、风景名胜区、森林公园、地质公园、湿地公园等保护和建设，不断提升生态功能。大力开展国家、省级森林城市和生态县乡村创建工作。建立健全环境信息公开制度、违法排污企业“黑名单”制度、违法排污行为有奖举报制度、企业环保诚信制度，严格落实生态环境保护责任追究和环境损害赔偿制度，切实解决“守法成本高，违法成本低”的问题，形成长效管理。加强宣传教育，营造全社会关注、支持、参与保护生态环境的浓厚氛围。

　　严格节能降耗。抓好工业、建筑、交通和公共机构节能，全面完成国家、省减排目标责任书项目20个，淘汰和关闭落后产能企业15家以上，单位工业增加值能耗下降4.5%。推动省级循环经济试点，实施一批节能技改和节能技术产业化示范项目，推进再生资源综合利用、分布式光伏发电等环保项目建设，逐步引进机动车使用甲醇或其他非污染、低耗能的动力。强化节能节约意识，大力推行绿色电子化办公。加快工业和生活污水处理厂及配套管网建设，提升运行效能。

　　严防环境污染。积极开展环境风险源排查，深入实施“三净”行动。着力改善空气质量，严格落实大气污染防治行动计划，推进中心城区3个空气自动监测子站升级改造，逐步开展PM2.5监测；重点整治建筑施工扬尘污染和机动车排气污染；全面实施陶瓷行业二氧化硫污染治理工程。加强水环境保护，完善赣江、袁河市界断面水质和饮用水源地水质24小时自动监测体系，实现所有饮用水源水库养殖“人放天养”；加大对袁河、锦江等流域污染行业企业的监管和整治，积极开展清淤疏浚。着力推进土壤污染综合整治，对重点污染物贮、运、用等各环节进行全程监管，及时消除事故隐患；大力推广绿色植保农药减量技术，推行畜禽清洁养殖和生态养殖，加大生猪养殖防治管理，在全市推广靖安垃圾无害化处理做法，切实减少农村面源污染。加大环保执法力度，强化环保、监察、公安等部门联动执法机制，严厉查处各类环境违法违纪行为。

　　（五）强化和谐稳定这一保障

　　积极回应群众的期待和诉求，大力创新社会管理和公共服务，确保社会和谐稳定。

　　完善社会保障。积极做好就业和再就业工作，吸引务工经商人员返乡创业，扶持下岗人员自主创业，引导高校毕业生自立创业，让每个社会成员有人生出彩、梦想成真的机会，力争新增城镇就业人数4.7万人，转移农村劳动力6.7万人。完善被征地农民社会保障政策，探索建立困难职工长效帮扶机制。大力推进保障性安居工程建设，新建保障性住房7721套，实施各类棚户区改造31112套。规范城乡低保管理，新建县级流浪乞讨人员救助场所4个。加快宜春市长红生态休闲老年公寓建设，新建奉新老年公寓康复楼，新改扩建乡镇敬老院18所，新扩建县市福利中心4所。推进“敬老文明号”创建。加强防灾减灾、应急救助和灾民保障，提高救灾科学化水平。完善残疾人康复托养和帮扶措施，积极开发适合残疾人就业的特色岗位。

　　繁荣社会事业。大力实施农村义务教育学校标准化建设工程，新建、续建和改扩建中小学17所，有效化解城区教育“大班额”问题。促进义务教育均衡发展，积极实施学前教育推进工程和普通高中资源扩充工程。支持宜春学院、宜春职业技术学院发展，办好国家开放大学（江西）试点学院。加快市人民医院（北院）、市儿童医院建设，健全新型农村合作医疗和重大疾病医疗救助等制度，继续推进卫生服务能力建设，探索开展医师多点执业，切实解决群众“看病难”问题。做好省第十四届运动会参赛工作，加快构建全民健身服务体系。加强人口计生工作，促进人口均衡增长，不断提高出生人口素质。加强文艺精品创作，实施文化惠民工程，做好第一次全国可移动文物普查。

　　创新社会管理。坚持系统治理、依法治理、综合治理和源头治理相结合，全面推进平安宜春建设。认真落实维护群众权益机制和重大决策社会稳定风险评估机制，从源头上预防和减少社会矛盾。坚持领导干部接访、机关干部下访、网上受理信访，推动信访秩序进一步好转。全面落实安全生产责任制，深入推进“打非治违”，预防和减少较大以上安全事故发生。加大引导和调解力度，构建工业园区和谐劳动关系。加强重大疫病防治和食品药品安全监管，保障公共卫生安全。健全突发公共事件应急管理机制，不断提高应急处置水平。深入开展党政领导在线交流活动，继续加大政务信息公开力度，推动新闻发布、专家解读常态化。加强法律服务和普法宣传教育，全面推广“奉新经验”，实施法律顾问制度，加快法治化建设进程。大力发展农村新型社区，健全农村留守儿童、留守妇女、留守老年人关爱服务体系。积极做好第九届村（居）委会换届选举工作，推进基层协商民主。加强国防动员、民兵预备役、人民防空和优抚安置工作，争创全省“双拥”模范城，推进军民融合发展。完善 “三网”体系建设，加强流动人口和特殊人群的服务管理，严厉打击违法犯罪活动，确保公众安全感继续保持全省前列。

　　各位代表，增进民生幸福、提升百姓福祉，是政府工作的永恒主题和最高使命。去年我们重点抓好了《政府工作报告》“双十”工作，74个子项目中，有72个达到或超过进度要求，2个因征地拆迁原因导致进度滞后。今年将继续按照“项目化、时间表、责任人”的要求，推进有关续建项目，重点抓好“10+1”工程，即十大工业项目、十大基础项目、十大农业项目、十大旅游项目、十大环保项目、十大科技项目、十大物流项目、十大返乡创业项目、十大民生改善项目、十大社会管理项目和争创一批城市名片，以项目的快速推进，谋求发展的升级、百姓的实惠。

　　各位代表，面对发展的重任、人民的期盼，我们将进一步加强和改进政府自身建设，努力建设负责任、有作为、人民满意的政府。强化担当意识。本届政府已步入届中的关键期，迎来干事创业的黄金期。全体政府工作人员要更加敢于担当，突破条框束缚，打破思维定势，不为困难所阻，不为风险所惧，不为杂音所扰，创造条件、破解难题，努力把干不成的事干成，把难突破的工作突破。弘扬务实作风。一分部署，九分落实，务实才能落实，落实才是本事。大力发扬钉钉子精神，驰而不息，久久为功，以踏石留印、抓铁有痕的劲头抓落实，向群众承诺的事，说一项做一项，项项兑现；对群众关心的事，定一条办一条，条条算数；与群众利益发生冲突的事，有一件改一件，件件纠正。深入开展党的群众路线教育实践活动，以实际成效取信于民。坚守廉洁底线。认真落实党风廉政建设责任制，严格履行“一岗双责”和廉洁自律各项规定。自觉接受人大法律监督和政协民主监督，强化审计监管、行政监察，加强财政大额资金管理，严肃查处各类违纪违法案件，切实做到干部清正、政府清廉、政治清明。

　　各位代表，站在新起点，踏上新征程，我们有信心、有决心、也有能力担负起人民赋予的光荣使命。让我们在市委的坚强领导下，锐意进取，真抓实干，为推进文明幸福宜春建设、确保与全国全省同步全面建成小康社会而努力奋斗！
